# Characterization of the Promoter Regions of Two Sheep Keratin-Associated Protein Genes for Hair Cortex-Specific Expression

**DOI:** 10.1371/journal.pone.0153936

**Published:** 2016-04-21

**Authors:** Zhichao Zhao, Guangbin Liu, Xinyun Li, Ji Huang, Yujing Xiao, Xiaoyong Du, Mei Yu

**Affiliations:** 1 Key Lab of Agricultural Animal Genetics, Breeding and Reproduction of Ministry of Education, College of Animal Science and Technology, Huazhong Agricultural University, Wuhan, 430070, China; 2 College of Informatics, Huazhong Agricultural University, Wuhan, 430070, China; 3 College of Animal Science, South China Agricultural University, Guangzhou, 510642, China; Sanford Burnham Medical Research Institute, UNITED STATES

## Abstract

The keratin-associated proteins (KAPs) are the structural proteins of hair fibers and are thought to play an important role in determining the physical properties of hair fibers. These proteins are activated in a striking sequential and spatial pattern in the keratinocytes of hair fibers. Thus, it is important to elucidate the mechanism that underlies the specific transcriptional activity of these genes. In this study, sheep *KRTAP 3–3* and *KRTAP11-1* genes were found to be highly expressed in wool follicles in a tissue-specific manner. Subsequently, the promoter regions of the two genes that contained the 5′ flanking/5′ untranslated regions and the coding regions were cloned. Using an *in vivo* transgenic approach, we found that the promoter regions from the two genes exhibited transcriptional activity in hair fibers. A much stronger and more uniformly expressed green fluorescent signal was observed in the *KRTAP11-1-ZsGreen1* transgenic mice. *In situ* hybridization revealed the symmetrical expression of sheep *KRTAP11-1* in the entire wool cortex. Consistently, immunohistochemical analysis demonstrated that the pattern of ZsGreen1 expression in the hair cortex of transgenic mice matches that of the endogenous *KRTAP11-1* gene, indicating that the cloned promoter region contains elements that are sufficient to govern the wool cortex-specific transcription of *KRTAP11-1*. Furthermore, regulatory regions in the 5′ upstream sequence of the sheep *KRTAP11-1* gene that may regulate the observed hair keratinocyte specificity were identified using *in vivo* reporter assays.

## Introduction

Due to the favorable properties of wool for use in textiles, the improvement of wool quality (such as the fiber diameter, length, strength and elasticity) is important for the sheep industry. The wool/hair fiber that is produced by the wool/hair follicle bulb is composed of the cuticle, cortex and sometimes a central medulla. The primary structural proteins of hair fibers are the hair keratins and the keratin-associated proteins (KAPs). Hair keratins produce the keratin intermediate filament (KIF), which is surrounded by matrix KAPs. The KAPs are the major component of the matrix around the KIF and were demonstrated to bind preferentially to hair keratins through extensive disulfide bond cross-linking or intercellular connections via desmosomes to give rise to the rigid hair shaft. Therefore, the KAPs play an important role in determining the physical properties of wool/hair fibers [1–4].

To date, a total of 30 mammalian KAP subfamilies have been characterized and were found to exhibit molecular diversification within mammals, yielding a variety of hair phenotypes [5]. KAPs are typically encoded by genes that consist of a single exon and have been classified into three groups: high glycine-tyrosine (HGT), high sulfur (HS, <30 mol% cysteine content) and ultra-high sulfur (UHS, >30 mol% cysteine content) [1]. Beyond a small number of KAPs that are expressed in the hair cuticle or medulla, the expression of most KAPs was found to be predominantly restricted to the cortical region of hair fibers. Although the expression pattern of KAPs is region-specific within the three structural compartments of hair fibers, variations in the degree and region of mRNA or protein expression were also revealed among individual KAPs. For example, by using *in situ* hybridization analysis, Rogers et al revealed that the HGT *KRTAP* genes *KRTAP7*.*1*, *KRTAP8*.*1*, *KRTAP19*.*1*, *KRTAP19*.*2* and the HS *KRTAP* gene *KRTAP11*.*1* exhibited strong expression within the three structural compartments of human hair fibers in comparison to other *KRTAP* genes investigated in the study [6]. Of those genes, *KRTAP7*.*1*, *KRTAP19*.*1* and *KRTAP19*.*2* were expressed in the upper portion of the hair cortex, *KRTAP8*.*1* was detected in the lower cortex, and *KRTAP11*.*1* was strongly expressed in the late matrix and the entire cortex of human hair fibers. Meanwhile, the activation of different group of KAPs occurs in a sequential manner as the follicle bulb cells rapidly differentiate into either cortical or cuticle hair keratinocytes. It has been reported that in Merino wool follicles, hair keratin genes were activated first and appeared in the cortical region, followed by the HGT *KRTAPs*, which appeared in half of the cortex, while the expression of the HS and UHS *KRTAPs* was observed late in the process in the complementary half of the cortex [7, 8]. Taken together, the expression of *KRTAP* genes is tightly regulated and exhibits a complex pattern in which different *KRTAP* genes are activated at different stages of fiber formation and in different regions of the fibers. These findings suggest the existence of different transcriptional hierarchies that guide these unique sequential and spatial expression patterns and highlight the importance of understanding the mechanism by which the differential expression of *KRTAP* genes is regulated.

To date, the number of keratin and *KRTAP* genes for which transcriptional elements or factors involved in governing tissue- or cell-specific and differentiation stage-specific expression have been characterized is limited, and most of those genes are keratin genes. Two epithelial keratins (K5 and K14) are expressed as pairs by the mitotically active basal keratinocytes in most stratified epithelial tissues, such as the epidermis [9, 10]. The regulatory elements that direct proper epidermis-specific expression in vitro and in transgenic mice were illuminated for the human *KRT5* and *KRT14* genes, respectively [11]. *KRT5* and *KRT14* exhibited layer-specific regulation in the epidermis, with the expression of the two genes occurring at a high level in basal cell layer keratinocytes but being repressed in keratinocytes of the suprabasal compartment. A further investigation revealed that the coordinated activation of the proximal promoter region and the POU domain factor Skn-1a is important for *KRT14* promoter repression in suprabasal cells [12]. Moreover, a series of in vitro and in vivo studies defined the role of a minimal promoter containing the LEF-1 binding site and factors such as Sp1 in the regulation of *KRT2*.*10* transcription in the hair follicle cortex [13, 14]. Since that study, the functions of a number of genes that are expressed endogenously in the epidermis or the stratified epithelium were characterized using the three *KRT* gene promoters in transgenic mice [14–16]. With respect to the *KRTAP* genes, McNab et al initially characterized a promoter region of the murine UHS *KRTAP* that could direct the appropriate expression of a reporter gene construct in transgenic mice [17]. In addition, a recent report revealed that a 5**′** upstream region containing an NF-kappa-B binding site may activate sheep *KRTAP6*.*1* expression in fetal fibroblast cells [18]. However, due to the restricted expression in the three structural compartments of hair fibers and the lack of suitable cell lines, the number of *KRTAP* genes with defined regulatory elements is small. Therefore, the objectives of this study were to isolate *KRTAP* genes that are highly expressed in the sheep wool follicle, characterize the associated cDNA sequences, detect the active promoter sequences of the isolated *KRTAP* genes using transgenic mice and then investigate the regulatory regions and factors involved in the observed tempo-spatial expression patterns.

## Materials and Methods

### Animal materials and sample collection

The animal experiments and procedures were carried out in strict accordance with regulations of the Standing Committee of Hubei People's Congress and approved by the Biological Studies Animal Care Committee of Hubei Province, P.R. China, and the Ethics Committee of Huazhong Agricultural University, P. R. China.

In order to investigate the regional expression patterns of the *KRTAP* genes in the three wool fiber structural compartments (cuticle, cortex and medulla), Tibetan sheep whose wool fiber contains all the three parts were used as the experiment animals. Three healthy (one male and two female), 1-year-old Tibetan sheep were obtained from the Agriculture and Animal Husbandry College of Tibet, China. All sheep were raised according to the sheep feeding standard of China. Animals were slaughtered with a rapid intravenous infusion of T-61 euthanasia solution. Fifteen tissue samples were collected. From those samples, fourteen tissues (skin, heart, liver, spleen, lung, kidney, rumen, abomasums, duodenum, rectum, muscle, lymph, testis and ovary) were immediately frozen in liquid nitrogen and then stored in a -80°C freezer for total RNA extraction. However, the wool follicle bulb tissue samples were homogenized in Trizol reagent (Invitrogen, Carlsbad, CA, USA) immediately after collection and then centrifuged at 12,000 rpm at 4°C for 10 min. The supernatant was preserved and stored at -80°C.

### Analysis of gene expression profiles

Total RNA was isolated from each tissue using the Trizol reagent (Invitrogen, Carlsbad, CA, USA) in accordance with the manufacturer′s instructions. The PrimeScript™ RT reagent kit (Takara Bio Inc., Dalian, China), which contains the oligo dT and random primer mix, was used for the first-strand cDNA synthesis. The mRNA expression levels of *KRTAP3-3* and *KRTAP11-1* in fifteen tissue samples from Tibetan sheep were evaluated via RT-PCR, and the mRNA expression levels of those genes in wool follicles and skin were further measured using qRT-PCR. The employed primers (*KRTAP3-3 F/KRTAP3-3 R* and *KRTAP11-1 F/KRTAP11-1 R*) (**S1** Table) were designed based on conserved regions between the bovine *KRTAP3-3* (GenBank: BC114204.1) and ovine *KRTAP11-1* (GenBank: HQ595347.1) sequences, respectively. The qRT-PCR assay was performed using the SYBR Green All-in-One QPCR Mix (Genecopoeia, Rockville, MD, USA). The relative gene expression was analyzed using the 2^−ΔΔCT^ method [19]. The ovine 18S ribosomal RNA gene (**S1** Table) was used as an internal control.

### Molecular cloning of the *KRTAP3-3* and *KRTAP11-1* cDNAs

The total RNA extracted from the skin was used to clone the *KRTAP3-3* and *KRTAP11-1* cDNAs. The CDS sequences of the bovine *KRTAP3-3* gene and the ovine *KRTAP11-1* gene were BLAST searched against the ovine genome to obtain the 5′ and 3′ untranslated regions of the sheep *KRTAP3-3* and *KRTAP11-1* genes, respectively. The primers (**S2** Table) were designed based on the 5′ and 3′ untranslated region sequences. The purified PCR products were sequenced by TSINGKE Biological Technology Co., Ltd. (Beijing, China).

The molecular weight (MW) and isoelectric point (pI) were calculated using the Protparam tool (http://web.expasy.org/protparam/). The phylogenetic trees were constructed by using the MEGA6 software (http://www.megasoftware.net/).

### Construction of expression vectors and generation of transgenic mice

Genomic DNA was extracted from Tibetan sheep skin using the TIANamp Genomic DNA Kit (TIANGEN, Beijing, China) following the manufacturer′s protocol and was used to clone the 5′-upstream regions of *KRTAP3-3* and *KRTAP11-1*. The 6,404 bp and 5,954 bp sequences upstream of the translation initiation sites (ATG) of *KRTAP3-3* and *KRTAP11-1*, respectively, were obtained. The KRTAP3-3p F primer contained an *Xho* I site, and the KRTAP3-3p R primer included a *Sac* II site. The KRTAP11-1p F/R primers contained a *Sac* II site and a *BamH* I site, respectively (in lowercase, see **S3** Table). After amplification, digestion and purification, the two promoter sequences containing 5′ flanking/5′ untranslated regions were cloned into the pZsGreen1-1 vector (Clontech, Mountain View, CA, USA), which is a promoterless vector encoding the green fluorescent protein ZsGreen1. The constructed vectors used for transgenic mouse generation were named KRTAP3-3-ZsGreen1 and KRTAP11-1-ZsGreen1. The transgenic mice were created through a commercial service (BGI Art Biotechnology Co. LTD., Shenzhen, China). In brief, the constructed KRTAP3-3-ZsGreen1 and KRTAP11-1-ZsGreen1 vectors were linearized using the restriction enzymes *Xho* I and *Afl* II, respectively. After extraction and purification using the phenol chloroform, the concentrations were all 18 ng/μl. The transgenes were microinjected into the fertilized Kunming White mouse eggs and were subsequently re-implanted into the pseudo-pregnant females. The mice were housed in the Laboratory Animal Research Center at Huazhong Agricultural University in Wuhan.

### Validation of transgenic mice

First, genomic DNA was isolated from tail biopsies from founder mice using the TIANamp Genomic DNA Kit (TIANGEN, Beijing, China) according to the manufacturer′s protocol. The genomic DNA was used to screen the transgenic mice. PCR primers (**S3** Table) were designed based on the promoter sequences of the KRTAP3-3-ZsGreen1 and KRTAP11.1-ZsGreen1 constructs. Second, the sheared dorsal hair from PCR-positive founder mice was visualized using a fluorescence inversion microscope system (NIKON, Japan) to detect the green fluorescent signal. Founder mice with green fluorescent signal-positive hair were used to establish the transgenic line. Third, the offspring that showed a strong green fluorescent signal in the hair were anesthetized and imaged using a whole-body fluorescence imaging system (Maestro In-vivo Imaging Systems, CRI, Inc., Woburn, MA, USA) to detect the expression of the ZsGreen1 protein. Then, the hair and eleven internal organs were removed from the mice and imaged using the same system. A negative mouse was as a negative control (NC).

### *In situ* hybridization and immunohistochemical analysis

Sheep skin containing wool follicles was collected from three 1-year-old Tibetan sheep during the anagen phase and fixed in 4% paraformaldehyde for 24 h. Then, 5 μm paraffin sections were hybridized with a custom-designed QuantiGene ViewRNA probe against sheep *KRTAP11-1* (Affymetrix). Nonradioactive ISH (In situ hybridization) was performed using the QuantiGene ViewRNA FFPE Assay (Affymetrix/Panomics), according to the manufacturer’s instructions and a previously described method [20]. The hybridization signals were recorded using an Olympus BX53F light microscope (OLYMPUS, Tokyo, Japan).

Immunohistochemical analysis (IHC) was performed to determine the presence of ZsGreen1 driven by the sheep *KRTAP11-1* promoter in mouse hair follicles. Skin containing hair follicles was collected from green fluorescent signal-positive mice during the anagen phase (postnatal day 14) and embedded in a mixture of beeswax and paraffin. IHC of fresh sections was performed according to a previously described method [20]. The primary antibody was the rabbit recombinant Living Colors® full-length ZsGreen1 Polyclonal Antibody (1:100, 632474; Clontech, Mountain View, CA, USA), and the secondary antibody was a biotinylated secondary antibody (1:100, SA1022; Boster Corporation). All of the sections were stained immunohistochemically under the same conditions. Images were collected using an Olympus BX53F light microscope (OLYMPUS, Tokyo, Japan).

### Investigation of the transcriptional regulatory region of the *KRTAP11-1* promoter

First, 5′-RACE (rapid amplification of cDNA ends) was performed to determine the transcription start site of the sheep *KRTAP11-1* gene. Total RNA isolated from the skin was used for the first-strand cDNA synthesis, according to the manufacturer′s instructions (SMART^TM^ RACE cDNA Amplification Kit, Clontech, Mountain View, CA, USA). A reverse gene-specific primer designed based on the coding sequence was used to amplify the 5′ end of sheep *KRTAP11-1* (**S2** Table). The PCR products were cloned into the pMD19-T vector (Takara Bio, Inc., Dalian, China) and sequenced in two directions.

Second, the Promoter 2.0 predictor server (http://www.cbs.dtu.dk/services/Promoter/) and Promoter Scan (http://www-bimas.cit.nih.gov/molbio/proscan/) were used to analyze the core promoter of *KRTAP11-1*. The transcription factor binding sites within the 5′-upstream region of the *KRTAP11-1* gene were predicted by using two databases: TRANSFAC (http://www.biobase-international.com/product/transcription-factor-binding-sites) and Matinspector (http://www.genomatix.de/solutions/genomatix-genome-analyzer.html).

Third, genomic DNA from the skin of Tibetan sheep was used to amplify the ovine *KRTAP11-1* 5′-deletion fragments. The PCR fragments were generated using a common reverse primer that included a *BamH* I site and ten different forward primers that contained a *Sac* II site (**S4** Table). Each PCR product was digested separately with the *Sac* II and *BamH* I restriction enzymes and cloned into the pZsGreen1-1 vector (Clontech, Mountain View, CA, USA). The positive clones were sequenced by TSINGKE Biological Technology Co., Ltd. (Beijing, China).

Finally, sheep ear fibroblast cells (SEF, BGI Art Biotechnology Co., Ltd., Shenzhen, China) were grown in DMEM medium (HyClone, Thermo, USA) containing 1% PS and 10% FBS (Gibco, Life, USA). Human hair outer root sheath cells (HHORS, ScienCell, San Diego, California, USA) were cultured in MSCM medium containing 1% penicillin-streptomycin, 5% FBS and 1% mesenchymal stem cell growth factor (ScienCell, USA). All cells were cultured at 37°C in 5% CO_2_. Transient transfection was carried out using the cells telex system according to the manufacturer’s protocol. Images were collected using a fluorescence inversion microscope system (NIKON, Japan).

## Results

### Identification of highly expressed genes in wool follicles

**Fig 1:**
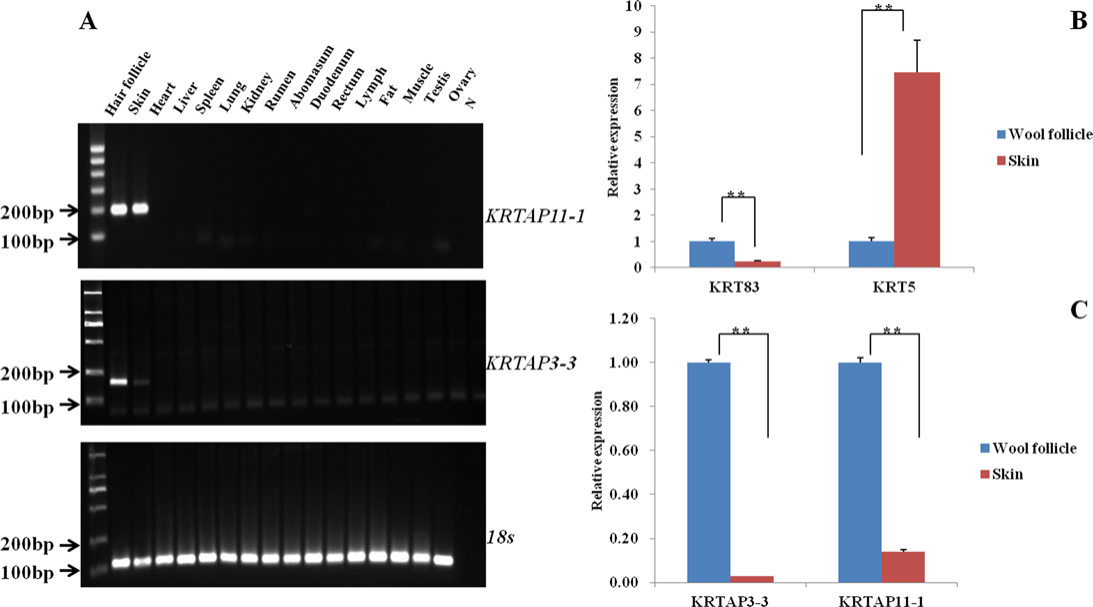
mRNA expression patterns of *KRTAP3-3* and *KRTAP11-1* in various tissues of the Tibetan sheep. (A) RT-PCR results of *KRTAP3-3* and *KRTAP11-1* in different tissues. Tissues: wool follicles, skin, heart, live, spleen, lung, kidney, rumen, abomasums, duodenum, rectum, muscle, lymph, testis and ovary. (B) qRT-PCR analysis of *KRT83* and *KRT5* in the wool follicles and skin of Tibetan sheep. (C) Relative expression of *KRTAP3-3* and *KRTAP11-1* in the wool follicles and skin of Tibetan sheep. Each experimental group contained three replicates, and qPCR was performed in triplicate for each sample. The data are presented as the mean± SD (error bars); **, P<0.01; Student’s test.

We previously analyzed the transcriptome profiles of the Tibetan sheep wool follicle bulb [21] and other four tissues (muscle, liver, spleen and lung; unpublished data). Comparative analyses of these data revealed candidate genes that were expressed at higher levels in the wool follicle bulb (data not shown). Subsequently, we investigated the expression patterns of these genes in fifteen tissues (wool follicle, skin, heart, live, spleen, lung, kidney, rumen, abomasums, duodenum, rectum, muscle, lymph, testis and ovary) using RT-PCR. The results showed that *KRTAP3-3* and *KRTAP11-1* mRNA expression was detected at high levels in the wool follicle and skin but was not detected in the remaining tissues (Fig 1A). Third, given that it is difficult to completely separate the wool follicles from the skin, the skin samples we used contained wool follicles. Therefore, we performed quantitative real time RT-PCR (qRT-PCR) to verify whether the mRNA expression levels of *KRTAP3-3* and *KRTAP11-1* genes were higher in wool follicles than in the skin. The *KRT83* and *KRT5*, which were demonstrated to be expressed specifically in wool follicles and skin, respectively, were considered to be the reference genes in this study [10, 14] (Fig 1B). We found that the mRNA expression levels of the *KRTAP3-3* and *KRTAP11-1* genes were significantly higher in wool follicles than in the skin, indicating that the observed expression patterns were consistent with that of *KRT*83 (Fig 1C). Thus, our data revealed strong expression of *KRTAP3-3* and *KRTAP11-1* in sheep wool follicles.

### cDNA cloning and sequence analysis of sheep *KRTAP 3–3* and *KRTAP 11–1*

**Fig 2:**
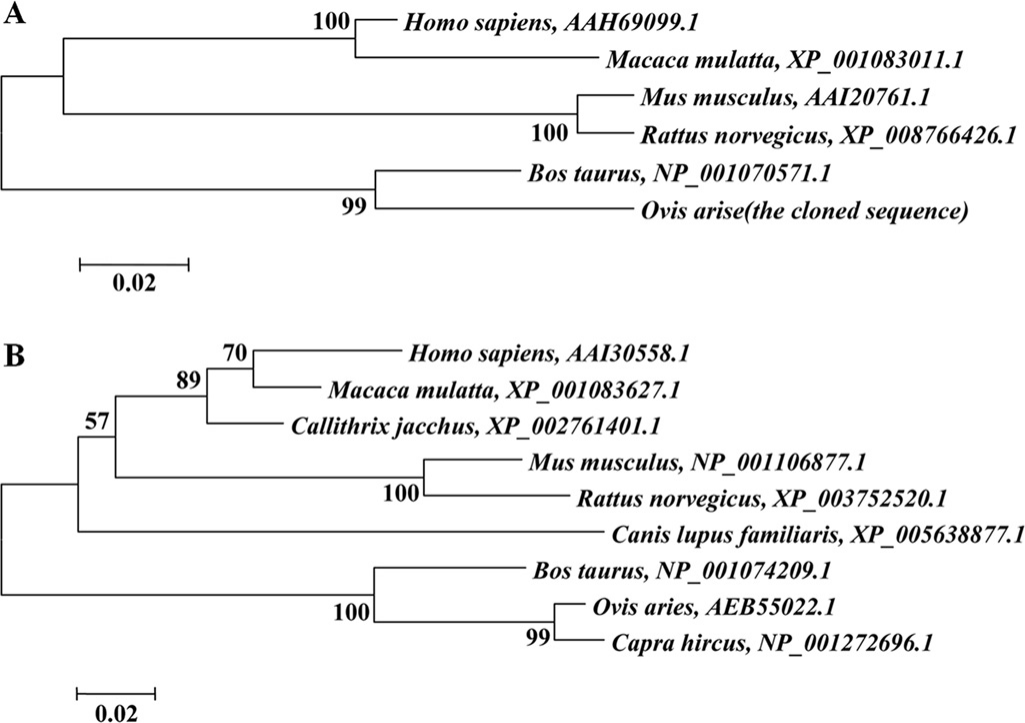
Phylogenetic tree based on the amino acid sequences of the KAP3-3 and KAP11-1 proteins from sheep and other species. (A) KAP3-3. (B) KAP11-1.

We isolated the cDNA sequences of *KRTAP3-3* and *KRTAP11-1* from Tibetan sheep skin (**S1** Appendix). A BLAST search of the Ovine Genome assembly v3.1 revealed that *KRTAP3-3* and *KRTAP11-1* were localized to sheep chromosome 11 and chromosome 1, respectively. The sequence analysis demonstrated that both of the genes contained a single exon and were well conserved across species. The derived *KRTAP3-3* cDNA sequence was 956 bp in length and included a complete open reading fragment (ORF) of 297 bp that encodes a putative 98-amino acid protein with a mass of 10.5 kDa and a theoretical pI of 5.99. The derived *KRTAP11-1* cDNA sequence was 900 bp in size and contained an ORF of 480 bp that encodes a putative 159-amino acid protein with a mass of 16.9 kDa and a theoretical pI of 8.38. The mammalian amino acid sequences of KAP3-3 and KAP11-1 available in the GenBank database were obtained for phylogenetic analyses. The results showed that sheep KAP3-3 and KAP11-1 were grouped with bovine KAP3-3 and KAP11-1, respectively, at a relatively high bootstrap confidence value, indicating that ovine KAP3-3 and KAP11-1 have the highest homology with *Bos taurus* and *Capra hircus* and the lowest homology with *Homo sapiens* (Fig 2).

### Generation and characterization of transgenic mice

**Fig 3:**
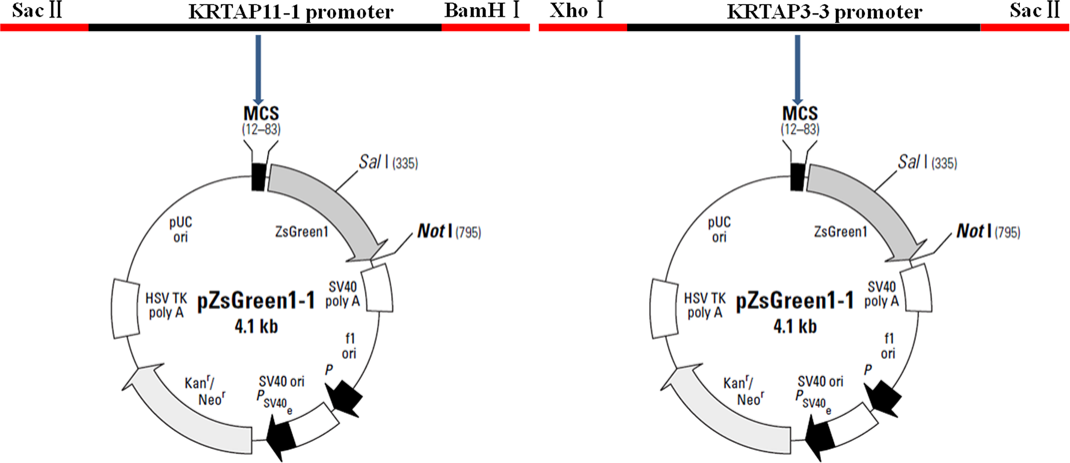
Structure diagram of the KRTAP11-1-ZsGreen1 and KRTAP3-3-ZsGreen1 vectors.

**Fig 4:**
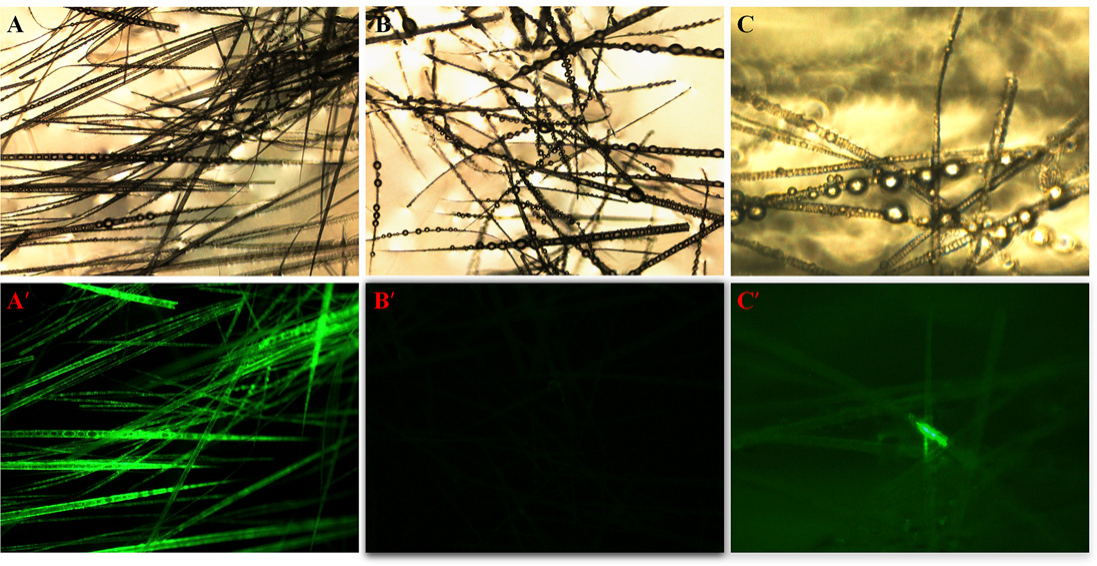
Expression of ZsGreen1 in sheared dorsal hair from a transgenic mouse. The hairs of the mice were examined under a fluorescence microscope. The upper and lower images were observed under white and fluorescent light, respectively. (A, A') The hair of the KRTAP11-1-ZsGreen1-positive mouse. (B, B') The hair of the negative control (NC) mouse. (C, C') The hair of the KRTAP3-3-ZsGreen1-positive mouse.

**Fig 5:**
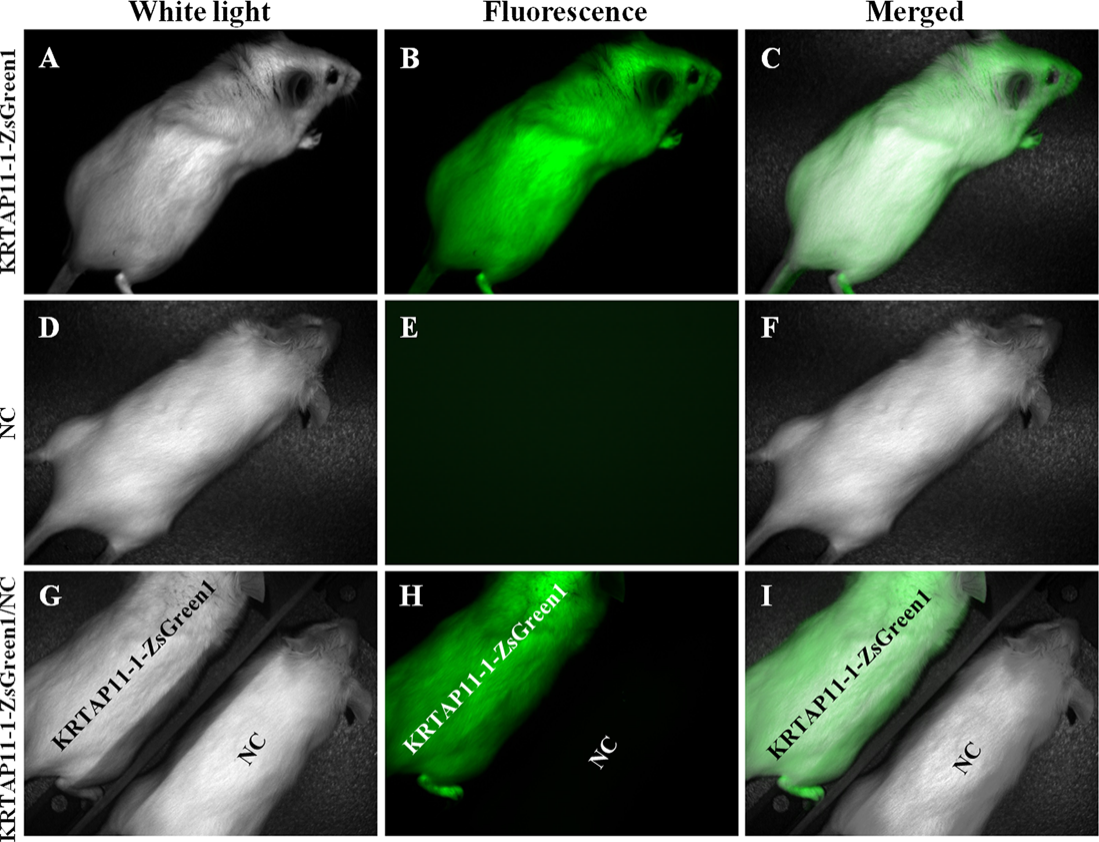
Fluorescence images of the KRTAP11-1-ZsGreen1 transgenic mouse obtained using the whole-body imaging system. (A) KRTAP11-1-ZsGreen1 transgenic mouse under white light. (B) KRTAP11-1-ZsGreen1 transgenic mouse under fluorescent light. Strong ZsGreen1 fluorescent signals were detected in the KRTAP11-1-ZsGreen1 transgenic mouse. (C) Merged picture (A) and (B); (D) Negative mouse (NC) under white light; (E) Negative mouse under fluorescent light. No ZsGreen1 fluorescent signal was found in the negative mouse; (F) Merged picture (D) and (E); (G) KRTAP11-1-ZsGreen1 transgenic mouse/NC mouse under white light; (H) KRTAP11-1-ZsGreen1 transgenic mouse/NC mouse under fluorescent light. The KRTAP11-1-ZsGreen1 transgenic mouse exhibited a strong ZsGreen1 signal under fluorescent light; (I) Merged picture (G) and (H).

**Fig 6:**
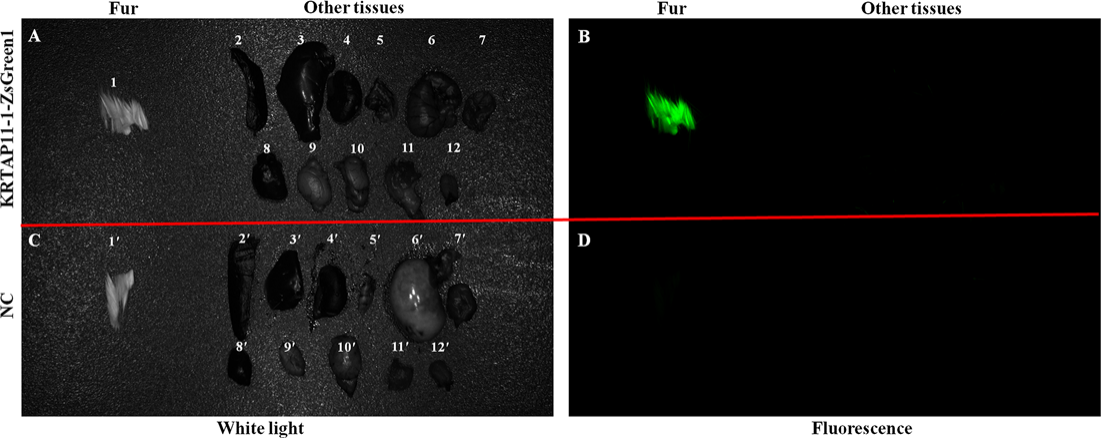
Fluorescence images of tissues from the KRTAP11-1-ZsGreen1 transgenic mouse. The tissues of KRTAP11-1-ZsGreen1 and negative mice were examined under white and fluorescent light. **(**A) The tissues of the KRTAP11-1-ZsGreen1 transgenic mouse under white light. 1: Fur; 2: Spleen; 3: Liver; 4: Kidney; 5: Lung; 6: Stomach; 7: Intestines; 8: Heart; 9: Testis; 10: Brain; 11: Muscle; and 12: Tongue. (B) The tissues of the KRTAP11-1-ZsGreen1 transgenic mouse under fluorescent light. Only the fur showed a strong green fluorescent signal. (C) The tissues of the negative mouse under white light. 1′: Fur; 2′: Spleen; 3′: Liver; 4′: Kidney; 5′: Lung; 6′: Stomach; 7′: Intestines; 8′: Heart; 9′: Testis; 10′: Brain; 11′: Muscle; and 12′: Tongue. (D) The tissues of the negative mouse under fluorescent light.

**Fig 7:**
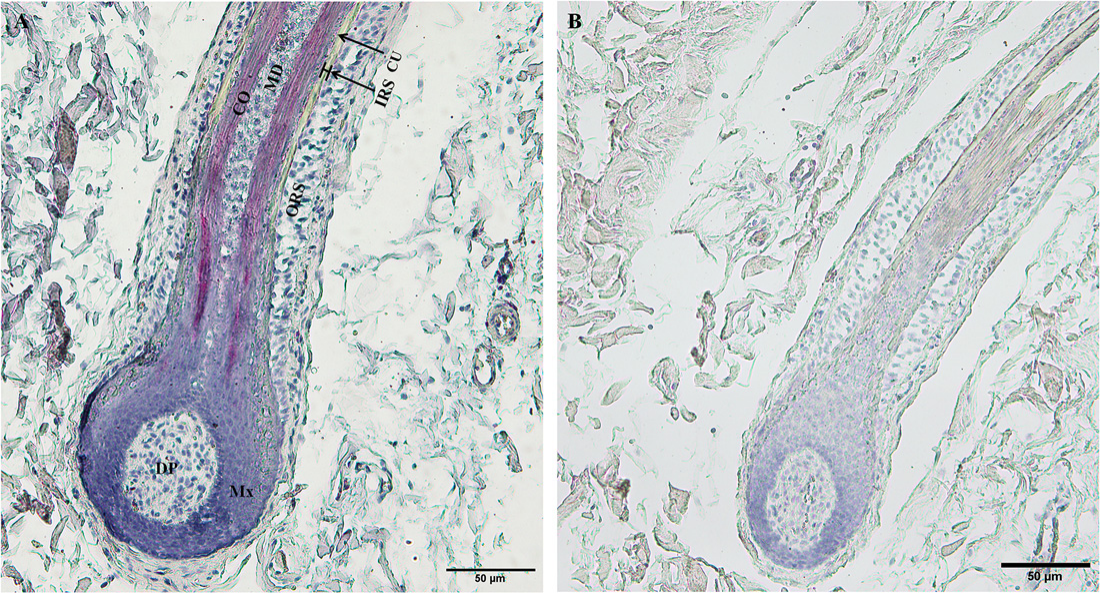
Localization of *KRTAP11-1* mRNA in wool follicles of the Tibetan sheep. (A) Red staining indicates that the expression of the *KRTAP11-1* gene is present in the hair cortex during the anagen phase. (B) Negative control. DP: dermal papilla; IRS: inner root sheath; ORS: outer root sheath; CO: cortex; CU: cuticle; MD: medulla. Scale bars = 50 μm (A, B).

**Fig 8:**
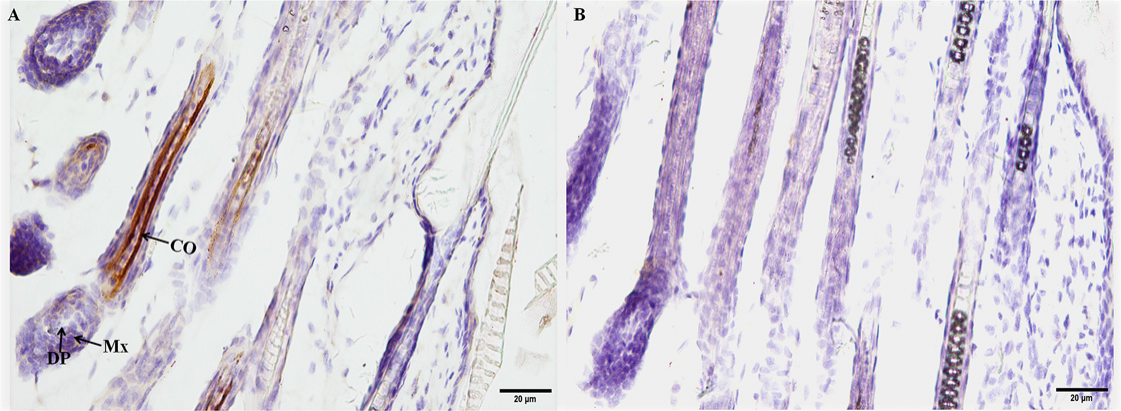
Immunohistochemical analysis of ZsGreen1 in dorsal hair from the KRTAP11-1-ZsGreen1 transgenic mouse. (A) Images of the hair follicles of the positive transgenic mouse were collected during the anagen phase (P14). A strong positive signal of ZsGreen1 was observed in the cortex of the hair follicle. No signal was detected in other cells of the hair and skin. (B) Negative control. Co: cortex; DP: dermal papilla; Mx: matrix. Scale bars = 20 μm (A, B).

To identify the upstream regions that regulate the transcriptional activity of the two genes, the 6,404 bp sequence upstream of the ATG of the *KRTAP3-3* gene and the 5,954 bp sequence upstream of the ATG of the *KRTAP11-1* gene (with the A of the ATG start codon numbered as +1) were isolated from sheep skin genomic DNA (**S2** Appendix). Then, each of the two isolated 5′ flanking/5′ untranslated sequences was ligated into the pZsGreen1-1 vector (Fig 3). Then the *KRTAP3-3-ZsGreen1* and *KRTAP11-1-ZsGreen1* transgenic mice were generated, respectively. The transgenic founders were characterized via a PCR assay using genomic DNA from tail biopsies. The sheared dorsal hair from the transgenic positive offspring mice was visualized under a fluorescent microscope. Strong ZsGreen1 fluorescent signals were detected in the sheared dorsal hair from *KRTAP11-1-ZsGreen1* transgenic mice. However, strong but scattered green fluorescent signals were observed in the sheared dorsal hair from *KRTAP3-3-ZsGreen1* transgenic mice (Fig 4). Therefore, only the *KRTAP11-1*-*ZsGreen1* transgenic mice were subsequently anesthetized and imaged using the Maestro in vivo imaging system to detect the expression of the ZsGreen1 protein. We found that the expression of ZsGreen1 was strongly visible along the hair of the transgenic positive mouse but undetectable in the negative mouse (Fig 5). In addition, fluorescence imaging also revealed that the green fluorescent signals were strong in the sheared dorsal hair but undetectable in the other 11 tissues investigated from the *KRTAP11-1*-*ZsGreen1* transgenic mouse (Fig 6). Furthermore, by using a nonradioactive *in situ* hybridization technique, we demonstrated that the *KRTAP11-1* mRNA was uniquely expressed in the cortex of the sheep hair shaft. No signals were detected in the inner and outer root sheath, the cuticle region or other adjacent skin tissues (Fig 7). We further defined the expression location of the ZsGreen1 protein in the hair follicles of the positive transgenic mouse. Immunohistochemical assays showed that the ZsGreen1 protein exhibited a strong signal in the cortex of the hair follicle (Fig 8). Thus, the localization of ZsGreen1 is consistent with that of the endogenous *KRTAP11-1* gene in sheep, indicating that the promoter sequence of sheep *KRTAP11-1* could drive ZsGreen1 to be expressed exclusively in the hair cortex in the transgenic mouse.

### Investigation of the regulatory elements in the promoter region of the *KRTAP11-1* gene in vitro

5′-RACE was performed using RNA from sheep skin tissue to obtain the 5′ UTR of the *KRTAP11-1* gene. The results revealed that the transcription start site was situated 51 bp upstream of the translation start codon (with the A of the ATG start codon numbered as +1), and an optimal Kozak consensus sequence (ACCATGG) was identified around the translation start codon. Subsequently, the minimal promoter region was predicted using the Promoter 2.0 and Promoter Scan software programs. Within the minimal promoter region, the TATA box and CCAAT box were located 31 bp and 253 bp upstream of the transcription start site, respectively.

**Fig 9:**
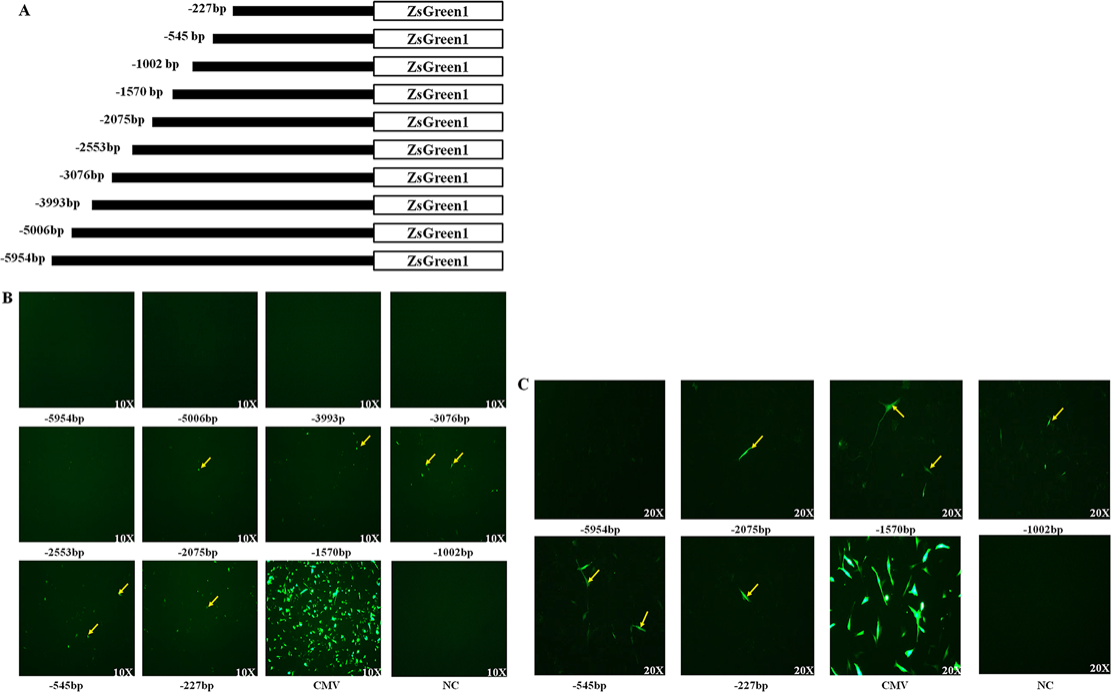
Transcriptional activity of a series of *KRTAP11-1* promoter deletion constructs in cells. (A) The construction of the truncated promoter vectors. (B) Transfection of SEFs with different truncated promoter vectors. The GFP signal was observed in the regions of -1,570 bp, -1,002 bp, -545 bp and -227 bp. (C) Transfection of HHORS cells with different truncated promoter vectors. The signal was detected in the regions of -2,075 bp, -1,570 bp, -1,002 bp, -545 bp and -227 bp. The number under the picture shows the relative distance from the 5′ site of the truncated promoter to the sheep *KRTAP11-1* gene translation start site; CMV promoter and Negative control.

To investigate the regulatory element in the 5,954 bp upstream sequence of the *KRTAP11-1* gene, a series of progressive 5′ deletions were examined for their effects on reporter gene activity in SEF (sheep ear fibroblast) cells and HHORS (human hair outer root sheath) cells, in which the endogenous *KRTAP11-1* gene is not expressed. First, the ten resulting constructs were transiently transfected into SEF cells. As shown in Fig 9B, weak but obvious activity was observed with four constructs (*KRTAP11-1-ZsGreen1*(-1570bp), *KRTAP11-1-ZsGreen1*(-1002bp), *KRTAP11-1-ZsGreen1*(-545bp) and *KRTAP11-1-ZsGreen1*(-227bp)). The four constructs were subsequently transiently transfected into HHORS cells, and the activity of three constructs (*KRTAP11-1-ZsGreen1* (-1570bp), *KRTAP11-1-ZsGreen1* (-1002bp) and *KRTAP11-1-ZsGreen1*(-545bp)) was confirmed (Fig 9C). By using TRANSFAC and MatInspector, five consensus transcription factor binding sites (NF-kappaB (p65), activator protein 1 (AP-1), GATA binding protein 3 (GATA3), ALX homeobox (Cart-1) and octamer-binding transcription factor 1 (Oct-1)) were predicted to be present within the region between -1,570 bp and -545 bp (**S3** Appendix).

## Discussion

Hair fibers originate from the follicle bulb. The differentiation of follicle bulb cells into either cortical or cuticle hair keratinocytes is determined primarily by the activation of keratin and *KRTAP* genes in a sequential and spatial manner. In this study, the sheep *KRTAP3-3* and *KRTAP11-1* genes were demonstrated to be highly expressed specifically in the wool follicle bulb via qRT-PCR and cDNA cloning. Subsequently, to define the regulatory functional region located upstream of the translation initiation sites of the two sheep genes, we established two transgenic mice (*KRTAP3-3-ZsGreen1 and KRTAP11-1-ZsGreen1*) and found that the two isolated promoter fragments encompassing the immediate 5′ flanking/5′ UTR sequences could direct the exclusive expression of the ZsGreen1 protein in the hair. Compared to *KRTAP3-3*-*ZsGreen1* transgenic mice, a much stronger and more uniformly expressed green fluorescent signal was observed in the *KRTAP11-1*-*ZsGreen1* transgenic mice. Furthermore, the *KRAP11-1* gene was demonstrated to be expressed symmetrically in the entire cortex of wool by using *in situ* hybridization. Consistently, immunohistochemical analysis revealed that the 5,954 bp upstream sequence of the sheep *KRTAP11-1* gene also drove the expression of the green fluorescent protein in the entire hair cortex in transgenic mice. Moreover, in vitro analysis suggested that some common regulatory elements, including NF-kappaB (p65), AP-1, GATA3, Cart-1 and Oct-1, were present in the region between -1,570 bp and -545 bp.

The *KRTAP3-3* and *KRTAP11-1* genes both belong to the high sulfur group and are located on sheep chromosomes 11 and 1, respectively [22, 23]. *KRTAP3-3* is a member of the *KRTAP3* family, which consists of three protein-coding genes and one pseudogene in humans [24, 25]. We obtained a 956 bp cDNA sequence for *KRTAP3-3* from sheep skin tissue and found that the included 297 bp coding region exhibited 99% identity with the previously reported sheep *BⅢB4* gene [25], indicating that the sheep *KRTAP3-3* gene is a protein-coding gene. In addition, the expression of the *KRTAP3-3* mRNA and protein has been demonstrated to occur in the wool fiber cortex or hair [2, 26]. As the only known member of the *KRTAP11* family [27], the genomic sequence of the sheep *KRTAP11-1* gene, which consists of only one exon, was reported previously. Our results concerning the isolated *KRTAP11-1* cDNA sequence further confirmed that the *KRTAP11-1* gene was expressed in the sheep skin tissue. Furthermore, both the human and mouse *KRTAP11-1* genes were reported to be expressed in the entire cortex [6, 28]. We also found that the sheep *KRTAP11-1* gene showed uniform cortical expression and was clearly absent from the central medulla of the wool fiber. Taken together, our study revealed that, similar to their orthologs, the sheep *KRTAP11-1* and *KRTAP3-3* genes are strongly expressed exclusively in the cortical region of wool fibers.

To investigate the controlling sequences involved in the regulation of sheep *KRTAP11-1* and *KRTAP3-3* expression and their effects on wool quality, we investigated the activity of the isolated 5′ upstream sequences of the two genes in controlling the expression of the *ZsGreen1* gene in transgenic mice. The results showed that both sequences could govern proper *ZsGreen1* gene expression in the hair of the transgenic mice, but the degree of activity differed between the two constructs. The transgenic mice were generated via the pronuclear microinjection of constructs in this study. The two main disadvantages of the system are the variability of transgene expression, which is caused by random integration and the unpredictable number of copies that eventually incorporate into the host genome [29–31]. Thus, the scatter distribution of fluorescent signals along the hair of the *KRTAP3-3-ZsGreen1 *transgenic mice may be primarily a technical artifact. Additionally, the observed pattern may also be caused by the absence of the necessary controlling elements in the *KRTAP3-3*-*ZsGreen1* construct or by other regulatory factors, especially those that are functionally conserved between mammals. On the other hand, a strong and uniform distribution of fluorescent signals was evident in the hair of the *KRTAP11-1*-*ZsGreen1* transgenic mice. Further characterization demonstrated that the ZsGreen1 protein was expressed in the mouse hair fiber cortex, which is consistent with the expression pattern of endogenous *KRTAP11-1* mRNA in sheep wool fibers.

Due to the lack of a hair cortical cell line in which *KRTAP11-1* is expressed, SEF and HHORS cells, in which the endogenous *KRTAP11-1* gene is not expressed, were used instead to investigate the regulatory elements in the 5,954 bp upstream sequence of *KRTAP11-1*. Transient transfection assays showed that the region located between -5,954 bp and -1,571 bp was not active. However, detectable transcriptional activity was observed within the region between -1,570 bp and -545 bp (1,026 bp in length) in both cell types, which implied that this region plays a role in the ubiquitous expression of *KRTAP11-1*. The proximal 1,026 bp of the sheep *KRTAP11-1* gene 5′ promoter region contains putative regulatory motifs, including NF-kappaB (p65), AP-1, GATA3, Cart-1 and Oct-1 motifs (**S3** Appendix). Sequence comparisons revealed that the putative regulatory motifs were highly conserved across sheep, humans, and mice. The activation of the five transcription factors is not cell type-specific, and of the identified factors, NF-kappaB (p65) and AP-1 have been suggested to influence the expression of human *KRT6 *expression in HeLa cells or epidermal keratinocytes [32]. In addition, sheep *KRTAP6-1* is another gene with hair cortical keratinocyte-specific expression [33]. Similar to our results, Yang et al reported that the proximal 1.5 kb of the sheep *KRTAP6-1* gene 5′ promoter region contained an NF-kappaB binding site and was capable of enhancing the transcription of the reporter gene in sheep fetal fibroblast cells. In contrast, the sequences outside the 1.5 kb proximal promoter showed significantly decreased transcriptional activity [18], suggesting that the region plays a dispensable role [34]. Thus, our findings indicated the possibility that the regulatory elements required for the hair cortical keratinocyte-specific activity of the sheep *KRTAP11-1* promoter may exist outside of the 1,026 bp proximal promoter region. A recent study used an organ culture system to reveal that the mouse KAP11-1 protein may participate in the assembly of micro/macro fibrils and consequently contribute to the formation of the appropriate hair structure. That study also found an altered expression pattern of the KAP11-1 protein without impaired expression in the hair cortex in forkhead box N1 (*Foxn1*, also named *Whn*) *nu*de mice [28], indicating that the expression of the KAP11-1 protein is at least partially dependent on Foxn1 activity. Foxn1 is a transcription activator that is transcribed primarily in the hair cortex and cuticle and has been established to play a role in the regulation of hair keratin gene expression [35–38]. It is possible that Foxn1 is a cofactor responsible for the activation of KAP11-1. Thus, further characterization of the *KRTAP11-1* 5′ region in a transgenic system is needed.

In conclusion, we characterized an ~5 kb sequence of the 5′ upstream region of sheep *KRTAP11-1* that can strongly direct the expression of ZsGreen1 in the mouse hair fiber cortex, corresponding to the location of endogenous *KRTAP11-1* expression in sheep wool fibers. In addition, the regulatory region of the sheep *KRTAP11-1* gene that governs hair keratinocyte specificity was investigated in vitro. Our findings represent a first step toward understanding the role of sheep KAP11-1 in keratin intermediate filament assembly in the wool cortex. In addition, the characterized promoter of *KRTAP11-1* could be a candidate for targeting the expression of foreign genes to manipulate specific hair/wool traits.

## Supporting Information

S1 Appendix cDNA sequences of the sheep *KRTAP3-3* and *KRTAP11-1* genes. (DOCX)

S2 Appendix Promoter sequences of the sheep *KRTAP3-3* and *KRTAP11-1* genes. (DOCX)

S3 Appendix Transcription factor binding sites predicted by TRANSFAC and MatInspector. (DOCX)

S1 Table Primers used to investigate the tissue expression profiles of the two sheep genes via RT-PCR and qRT-PCR. (XLSX)

S2 Table Primers used to clone the *KRTAP3-3* and *KRTAP11-1* cDNAs. (XLSX)

S3 Table Primers used to clone the *KRTAP3-3* and *KRTAP11-1* promoters. (XLSX)

S4 Table Primers used to amplify the *KRTAP11-1* 5'-deletion fragments. (XLSX)
