# Supplementary material for: Characterization of the Promoter Regions of Two Sheep Keratin-Associated Protein Genes for Hair Cortex-Specific Expression
Source: PLoS One. 2016 Apr 21;11(4):e0153936. doi: 10.1371/journal.pone.0153936 (PMC4839604; doi:10.1371/journal.pone.0153936)
Supplement: S1 Appendix — (DOCX) [file pone.0153936.s001.docx]

**The cloned partial cDNA sequence of the sheep *KRTAP3-3* gene (5′-3′)**

TTCTCACCCCAGGAGACTCGTCTTCTTTAAACCAAATCAATAAAAAACACAGCTTCCCAACACC**ATG**GCTTGCTGTGCCCGCCTCTGCTGCAGCGTCCCCACCAGCCCAGCCACCACCATCTGCTCCTCTGACAAATTCTGCAGATGTGGAGTCTGCCTGCCCAGCACCTGCCCACACACAGTCTGGTTCCTGGAGCCAACCTGCTGTGACAACCGCCCCCCACCTTGCCACATTCCTCAGCCCTCTGTGCCCACCTGCTTCCTGCTCAACTCTTCCCAGCCCACCCCAGGCCTGGAAAGCATCAACCTCACAACCTACACTCAGCCCAGCTGTGAGCCCTGCATCCCAAGCTGCTGC**TGA**CCGACGGCTGCCTCACCCAGTGCCTGACAGAGTCAACCCAGAAGCTTTAGTGCTCACCTGTCTCAGTACCTGCGACTAATTATGTCTCCGCTTTCAAAGTTGGAACAAGGCATTATCACAGACAACCCTCACAAAAAACAAACCAAGAGACTTTCGATGGCCGTGTAGTGGACATCAGTGAACAAGGACAGCTGGAGTAGGTAGATGCCTACAGGTTTCCCAGCGTTGTTCAGTTCCTTCGTGTTAAATTGTATCTTTCTTTGGTGCTTTGGGAATTCTGTTTCCAGTCTTGAATGGCATCTTTCTGGAAATTGAGGAGCTTCTTCATGATTATTCTAATAAAGTTTACATCTCTGGCATAACATAAATGTCTATAGGTATTTCCATTTATTTTTGTTAACACATCGAATGTATTTCTTAACTCCAAAGGTCAGCATTTTAAGAAACTGAGAACATCTGCAGAACTGAAAAGAGTGGCAGTACAGACAAATACTTCTTGCTTTTGTTGTTCTTGTTGTTTTAAGAAAACTAAATGGTGTTGCATTATAAAATGGTCCAGGATATCAGCATAATTTCCCTGACTTGTCCTTCCTCT

**Note:** We cloned partial cDNA sequence of the sheep *KRTAP3-3* gene including the complete open reading frame (ORF from ATG to TGA ) ( the red ,bold front).

**The cloned partial cDNA sequence of the sheep *KRTAP11-1* gene (5**′**-3**′**)**

AGAACCAGCCTCAGTGAGTTACCCACATCTCTCCACCAGCACC**ATG**TCCTACAGCTGCTCCACAAGGAACTGCTCTTCCAGGCGGATTGGAGGAGAATACACTGTTCCAGTGGTCACAGTTTCTTCCCCGGATGCCGATTGCCTGAGTGGCATCTATTTGCCCAGCTCCTTCCAAACGGGCTCCTGGCTCCTGGACCACTGTCAGGAGACCTGCTGTGAGCCCACTGTTTGCCAGTCAACTTGCTACCAGCCAACTCCTTGTGTCTCCAGCCCTGTGCGGGTGACCTCTCGGCAAACCACCTGTGTCTCCAGTCCCTGTTCGACTACCTGCAGCCGGCCACTCACCTTTATCTCCAGTGGCTGTCAGCCTCTGAGTGGCGTCTCTACTGTGTGCAAGCCAGTGAGAAGCATCTCCACTGTCTGCCAACCGGTGGGAGGAGTCTCCACCATCTGCCAACCTACCTGCGGGGTCTCCAGGACGTACCAGCAGTCCTGCGTGTCCAGCTGCAGAAGAATTTGC**TAA**GTTCAGAAGCCCATGAGTGAATCAAGATTCCATGACCTGCCAGCTGCGTTTCCAGGATCTTCCGACATGCTGCCTGAGTGACTTCATTGCTGACCCCTGTTCTAACTGCCTGATTGCTGGCTGCCAGCCCTGAATAAGCCGCCTTTGGCAATCTAATATTTGGCCGGCACCAATCTTATTTTAAGGGTTTGATGACTGGTGGCATGTATACCTCTGGATGTTTCCAGAAATGTACCACTCACGCCCCAGTCTCTAAGGGTTTTGGCATGTTTTGACCTTGCTGCTTTGTCTTCTGGCTTCTGCTTTTGTGCCTTGGAAAAGGGAACTTGTCTTGCTCTGTGTTTCTCAATAAAACCTCATTACTTGGCATTGCAAAA

**Note:** We cloned partial cDNA sequence of the sheep *KRTAP11-1* gene including the complete open reading frame (ORF from ATG to TAA ) ( the red, bold front).
