# Supplementary material for: Characterization of the Promoter Regions of Two Sheep Keratin-Associated Protein Genes for Hair Cortex-Specific Expression
Source: PLoS One. 2016 Apr 21;11(4):e0153936. doi: 10.1371/journal.pone.0153936 (PMC4839604; doi:10.1371/journal.pone.0153936)
Supplement: S2 Appendix — (DOCX) [file pone.0153936.s002.docx]

**The cloned promoter sequence of the sheep *KRTAP3-3*  gene (5′-3′)**

CTTATTTGGCTGAGTAACCCTTTGGTTCATTTGGATAGATAGAGGTCATCTATTATCAAAATAGTAGTGAAATCTATAAGACCTCAGACTCTATTTTCTTGGTCATGTTGCTTCTTGGGTCGCATATCTTGTATTGGGCATTTTCATAGAGGAAAAATAACTTTTGATGGTTTTCCAATAAACTTTGTTTCTGTGGCAAACTGTGTTTTTCATTTATGTTAGAGTTTTTGGTATATTTCTCTCATTTGGGAGAATGGCACTGAAACATGTATAATATCATATAAGAAACGAATAGCCAGTCTAGGTTCAATGCAGGATACAAGATGCTAGGGGCTGGTGCACTGGGATGACACAGAGGGATGGTATGGGGAGGGAAGTCGGAGGGGAGTTCAGGATTGGGAACACATGTACACCCATGGCAGATTCATGTTGATGTATGGCAAAACCAATACAATATTGTAAAGTAAAATAAAGTAAAAATAAAAATTTTTAAAAAAAGAAAAAAGTGAATAAAATAAATAAATTTAAATTTTAAAAAATATTTTAAAAATAAAGGAAAGATAATTTTTAAAAAAGAATCTCAGAGGAATGACTAAAACTAGCTTGCTAACATGGCTTGCTAACTTTAAACTGCTTATCTTTATATTCACCCTCTTTTCTCTTCCTCAGACAAACACAATCTGGGGGTGAGACGTTTACTTACCTACACTTTGCCAGGTAGATCACATGTATTAACTCATGTAACCCTTCAGTTCAGTTCAGTTCAGTTCAGTCGCTCAGTCATGTCCAACTCTTTGTGACCCCATGAATCGCAGCCCGCCAGGCCTCCCTGTTCATCACCAACTCCCGGAGTTCACTCAGACTCATGTCCATCAAGTCAGTGATGCCATCCAGCCATCTCATCCTCTGTCGTCCCCTTCTTCTCCTGCCCCCAATCCCTCCCAGCATCAGAGTCTTTTCCAATGAGTCAACTCTTCACATGAGGTGGCCAAAGTACTGGAGTTTCAGCTTTAGCATCAGTCCTTTCAAAGAAATCCCAGGGTTGATCTCCCTCAGAATGGACTGGTTGGATCTCCTTGCAGTCCAAGGGACTCTCAAGAGTCTTCTCCAACACCACAGTTCAAAAGCATCAATTCTTTGGTGCTCAGCCTTCTTCACAGTCCAACTCTCACATCCATACATGACCACTGGAAAAACAATAGCCTTGACTAGACGGACCTTAGTCAGCAAAGTAATGTCTCTGGTTTTGATTATGCTATCTAGGTTGGTCATAACTTTTCTTCCAAGGAGTAAGCGTCTTTTAATTTCATGGCTGCAGTCACCAACTGCAGTGATTAGCATAAAAAGAAAAATGAAGCTTTAAGAGTGATTCAACATTCTCAAAGTCACAAAGTTATTAAAAAATTCCAGGATTCAGCTCCTTGTATATCTGTTTGACTTTAAAGCCCAATTCTTTCTATCACATCACAGAACCCCCAGTGCTAACAGAGCCAGAAATTTAATAAACACAGGTATGACATTAACTTACAATATGAAAAAGAAATAAAAGTATAAAAATCTTTAACAAAATCATCCAATCCAGCTGTAATGGACAAGGATATATATATAGGCTTATAGATTAAAAGTGGCAGATATTCAGTTCTAAGAACCGGGTCATATAGTGAGCCTACCATTTTGACTTCATTTTTCCTTGGAGAAAACTTACAGTGGTCCATTCTAGTAGTAGGGCAATAGGAATATCCTTCTCCCTGCCACAACACTACCCCAGTAATGAACAGAACAGCAGTATTTTCCAGGTGATTGTCTGAGGGCTAGAGCTAAAGTCTGATTAAACATGGTTTAATCTACTACTTAATTTTACTTCATTTTACCAACCTACTTTTTTTTATCTTGCACAGATTATTTTTCCCAGCCTTAGATTACATATACATAAATATATTTTAGAATAGATTATATAAAAGTATATATTAAATATTTATATATAACTATTAAAATATATACATTAAGGTACATGTCAAACTCTGATAAAGCATTGAGCTAGAACCAAAAGCATCCATGGGAAAAACAAAACGAGCAAGGAAGGGGAGAAGCAAATGCCTTTAAATAAATGTTTTTAAATGTCCTCATTGTCCCTGTCACTCTCTCCAGAGAAGAATTTCTCAGAGGATCCCACAAGTTTATTAGAAGTTCAACTTCGAATGAACAAAAGTTAGAAGCTGTAGGGTAGATGGAACACAATCCAGGACCCAGTCAACAAAGGATTTCGTCCATTTGCTCATTCACTTGGCCACCATTTCTTGCATGCCTCCCCTGTGCCAGGCTTCAAATCCATACTAACAGTATAAAGATTAAAGAAATGTTATTATCTAAAGTTCCCTCACCTTCCTCTCAAATCTGGTGAAGGAGACAGACAAGTTATTACAATCACAGCACAGGTGTTCCGTGTATGTTGTATGAATGTAATTAGAATGCCATGGAATCTCTGCCGTGACAGAGCTGTGAGCAGTATGCCAGGCAAGCACACAGACAGAGTAACTAAAACCCAGATTTATCTGGTGACTCAGTGTCAGGGTGAGGGACAGTTATGAAAAGGCTTCAAGCTATCCAAGCTAAGCATTAAGAGATGATGAGGCAGAATTTAAAAAAAAGAAGAGATGACGAGGTGGAAGAGGTGAAGAGCAGAACAGGAGCTTACTAACTCATGGACACCTTGAAGATGGGAGAGGCTGGGGCCAAAGAGAAAGAAGAAATGCAAAGACCCTGAAGTGGGCCCCCAGCAGGGAGGTTCAGGCTGAACCCTCGAAGCAGGGAGAAATTTGAAGCGAGAGACTAACAAGCATATTTTACTTCTTATTTCTGTTACTTCTTATGATCAATCTTGGTATCTTTACTGTCACATACACCCCTACTGACTGTAAAAAGGTGTTATTAAAACTGGGCAAGTTCTAGACTCACAGACATAGAGAACAGACTTGTGGTCACCAAGGGGAAGGGGAGTGGGGAGGGAAGGACTGGGAGTTGGGATTTGCAGATGTAAACTATCACATGTAGGATGATGAGCAAAACATTCCCACGGTATAGCACAGGGAACTATGTTCAGTATCCTATGATAAACCATAATATAAAACAACATTAAAAAAGAATGTCTGTGTATAACTGAGTCACTTTGCTGTACAGCAGAGATTGACACAACTATATTTCAAAAAAAACAAAAACCAGACAAGTTCGTGAGTGGGTGTGCATGTACGTGTGTGTGTAGGTTTTCAAACTGTTGAAATTCATCAAGGATCACCATGTTTTTCCTGCACTTGCTTGAAGGAAAAGGGTACCTTTTCTTTCTAAAAATAAAGTATGTTTAATATCTCTAACACATACTCTAAACTTACATTTTAAACCACATTAAAGGTAACTCAGTCCCAGTAGGTCCTATATTTAGTTCTTAACAAGTCGGTCACCAGGCTACTTAAGGTAAATCATCTTACCTGTCTAGGATTCATGATCCTAATTTAAAAACAAAGGAGAGTTATTCCCAGGTCTCTTAGACAATTGCAGAGAAAATAAACTTTAATGAAGTACAAGTCCTTTCAATAAAAGATGAGTTTATTCATAAACTTGGTATTTTATGATTTTCTAGTCTGTAATGCTAAGTCTTATTTCTAGTTCTAGAAACATACTATTCCACACAGAATAGCAACAATTCCTTTATCCTCACAAGGTTGATTCAAAATAATATGCATTCTAAGTAAGAGACTGATATTTTTCTGCCATGAAATTCTAACTGCAAGAGTCTGAAAACTCCTTTATAATACTTTTCTTTAAAATGTCAAAATACTTTCCTTCAGCTTATTTTTTATCCTTGGAACCCAGTCTTTTCTTTTCCTTCATAAATTATAACAACAGTAACTTATAATAATACATTATGACATAAGACGTATACGTATGACAAACCTTCCTCCAGACCCTAAGTTTCCTGAAAGCAGGGATGATGTTAATTTTGTTCACTTTTGTATAATCACAGTTTCTGGCATATGGTAGCAGCTCAATAAATACCTACTGAATTATTTTGTAAATACTGCAAAAAAATATTAATCTGTCCTGAGTAAAAGGGTGAGCAATTTGGCAGGTATGCATTCAAAGATTCATCTGGGGGAAATCCAGGGACATCAGTAAAGATCATGAACACTTTATTTCAAGCAGAGCCATAGAGAACTTTTTAAAATTTGAAATGTATTCATTTCTGTGCCAGCCATTTTCTCTAACTGTAGGTGTGAACTTTTAGGTAAATTTTGCTGTTTCCTGTAAGGTTGCTCTTTAACTTGGTTTCTCCGCCTTCCTCTTCCCCTGCGTTTGATCTGTAACTTGCTTAGATAATGACAATAATTTGCCTTTGCATTGCACTTCATCTATGGATTTTAATCCATTTTTATCTCATTCGAGCCTCACAATAATCTCCAGAGTTGAAGAATGTAGATATAAGTATCCTTATTTTACAGAGGAGAAAGATGAGCTTCCAAAAAGTTAATTGCTTGCCATAGCAAGAAAGGTAGCCAGCAGTACAGTGACCAATTGTAACTCTTAACGGGGTCCTCTTGAATCCACACCCTCTGCAGGAAAAAACCACACACTTTCCCACCATCATGCACTGAGATACAGGTAGACTTCAAGGAAGAGAGGAGAACCATGACTTGAAGGATGAGTGAATCCAAGCTGAGAGGAAATGGTTTGAGTTGTCGGGATGACCCTGGACAGGAGTCTGCACTGTGCTCGCTCTTTATGCTGGACTATGCTTGAAGTCTAAATGGTCTACTGGAACTAGAATACAGGTCTTCCTTACCACAGCTGCATTGCACTTGGGGAGAAAACGTACTACTTTGGGGGAAAAAAAAAAACAATGAACAAAATCGATTGTGGATAGATTAACATAGGTCTTTAACTTCGCTTTCCATTGATAGGAATATTGGTAGTGAAATTATTCTTCTACTTAGTGTTTAGATATAAGCTAACAATTCAACTTTCCCACACTCGGTAGAGAAAGAAACAAAACAAGCCAGTTTTCGACAGAAATATACATGGCCAATGTGTATATGAAGGAATGTTTTAACCTAATAGTTATGAAAGAAATACAAATACTGTAACAATGGAATTTCATTTTCTCCTGACAAATTGACACACACTTTTTAAAATAAAATATCCATCAAAGTATACAGAAACAGGAATTTCCAAATAGCAGTTGGGAATATGTAAATAAGAAAAAGTGGCTAAATAAAATGATTCTAGAGAGAAGTATGGCAATGAATATCAAAGGTCTTAAAAATTAATTAACATTTGCTCCAATAATTCTATCCAATAATCAGTCCTCTTTCTGAAATGATCAGACACGTGCATAAAGATTTATGTACACAGTTGTTACTATTGCCACAATTATCTTAGAAGAAAAGTTGCAAAGCATTTAAATGATTAATACATGAGATTTCTAAGCAATTTAGAAGTACAACTATGTGATGAACACTAGGAAATACTTAAAATAATGTTATCAAATATAGTTAAGAATATAAGATCATGAAAATATATACTTAAGTGAAAATGCAAGTTATGGAATGGTATATACAACGTGATTCCAGTGGCATCATAAAATGATAAGCAAGTATATAGAAAAATGGCTAGAGGGATATATATAAACACTGATAGAGAATTTCTCTTAACGATAACATGTTGTGGATAATTTCTTTATCCATTTCTATATAGTCTGAACTTTTTAATGAATATGCAATATTTCACAACAAAAATGGGATAATAGTGTGGGTCTCCATATTAATGTAAATTAGATCTTCAAAAATCCCAACACTACAATGCTACTAATACAATGTTACATGTATATTATACATCAATTAAAAGTCAGTTAATTTATTAAAGTCCCCAAAGCTAAAAGAGAGTTACTAAAAATAAATTATCTTTAAAAGAAAAATAGTTCATCAAACCAATTGATAACAGATAAAGGTAAAGAAAATAAAAACTGACCAGTACGAGGGAAAATACCTACACCCTACAGCTGTACTGTCACGCATATTCCCCATCAGGGCTGATGCTGGAATTATAATAGCAGAAGCCACTGACGATACCTCATAAAGCTTGTGTTCCAGTGACAAATAGCAAGAGTAATTTGTTCAGATTTAGCAGTGAACTCAAACAAAGGTTAATTAGGAAAATAAAGACTCTTTACAAGCATCTAAATGTCAAACCAGTCAAAGGATAATGGGTACACTAACATAAACAACAACAGGAAAGAAGCCTTCCGGTTGGACCAACACCCACTGGAGAGGGTATATAAGAGCCTCAGAGCAGAAAGAGACATTCTCACCCCAGGAGACTCGTCTTCTTTAAACCAAATCAATAAAAAACACAGCTT

CCCAACACC**ATG**

**The cloned promoter sequence of the sheep *KRTAP11-1* gene (5′-3′)**

CCCCTGGATAGAAAGATCAGACCAAGAACAGTTGCATTGAAAGGGCATCAGCACAGGTCAGCTAGGTGATGCTAAAGACAAGATTTACCTTATACTTCTTTCTCTCACATAGCTCCTATTAATAGCATCCTTATCCTCCTGGGTACCAAGGAGACCCTGAATAATTCTGTATCAGGGAAGGATAATACACTTGTGTGACTTTGGGCCTTCTCTCTGGCCCTATACATAAACGTTACTCACGTCTGGTCAAAGGCGGGTTCACTTCTTGTTACTTGAAAGACTGGGACTGGAGCACATACCAGCTACACATGTGATATCCTGCAAGAGAGTTTGTTTGGTCTCCAAACTTGGGAATCTACTTACTTTTATTGAGAGCATCCTGATTCTGATCTCCAATAAAAGCATTCGTGAAAAAATACATGATTTTGAAAACAGGCATGAAGAAAGAGAATGGCAGTTCAACTTTGGATTTTTCAACCTTACATTATCCTCTGAGGATTTATTTTTCTACAAATCTTGTTGCTGGTGATTGCTATTTTCAAGTTCTAAAAGCAGCAACTATTTTGCTGGTCTTTGCCTGCTTCTTTCACTTTTCTGCTTTCTGCCTTACATTTCCCTCCAGCCCCAAAGATTCAAACCTTTGTATGATTGACGCTTTTTGAAAAGAGTTTGGAAAATGAATTTCAGCTCAGTTTTGGTGCAAATCACTCAGCCTTTGGGGGGTTTATAATGTCTTGGTTAATAAGAATTCTGGAGATTCAAGCTGGAGGTCTGTGTGAAGTTGGGAAAAAATGGCAGAACAATTCAGCCAAGATGTATTTTGTTTTTAAAGCAGTCATTATGTTCTGTCCTTAGAATTTTTCAATTCCCTTTTTATGTTTTTTAAGATAGAATGAATAGCTTTCATTTCAGAGTATGAGCATCCTGGAAAAGTGCAGGATGACAAATTCTAATTTTATGTTTATTAATTTTTCAGCAAGTTTAATATTGCTCCATTCGATGAGACTAGGTGATTTTTTTTTGAACCAGCATTGATAAACATTATTACCAATTTTGTGATAAAAAATATCCTTCCTGCATGTCAAAACTTCAGCCTCCCACACATGCTTAATTCTTTTTACTCTCAAACACTTGGCCTTGTCAGTGATTGCTGTGATTCAGTTTTGAGTGAAAGAATTGCTCATTTCCAGTCTGTGATAATTATTGTATAAGGCTTAGATTGTTCTATACAAACGAGAGCAAATTACCCATCAAGGATGTAGATAATGGGTAGTAGTACTTTAATCTCCTCTCATCTTCTCTCATCATATACTCCTCTCACTTCACACCCTGCAGACACAGGAATAATCTTTGCAAACCTTTTCGCTGACTTGAAGTTTGATATTGCATATAACTATCTCGTGATAGCTGCTGGATGCAAAATTTTTTCTTACATTCCAATGAAAGGATGATTTATTGATTTCACTTAGCAACACTGTAGACTTAGAATCATGTCTTCAAGCTTCATGTTGCTGTCAGTGCCTATTTCTTTTTTCTTTTGGCAAATTTTAGCATTGTTTTAATTAAGGAACATAGCTGTTTGAAACAGGAGATTTTAAGCTAGTTGAATAGAAGCAAAGTGAAAGATTCCTCTATTAGGATAGCTGCTATGAATAAAATCCTTACTATGTGGATTTGAATTCCATTGTAAATTTGGCACTAAACAACACTGGTCTAAGCCAAAAATTTCTCATTTTTGTTTAGAAGCCCTGAACATTTTCTTTCACATTTAAGGAGGGGAAAGGGAAGAAAAGGAAAAGGGGAAAAAAAGCACTCTATAATAGAATCTCATTAGCTTGATAATGGGTATGCTGATTGTAACTTGGTACTCCCTAAACAATTTCAGATAATGGCAAACAACTTAAAATGTAGGTGAGATGTGAATTTGCAAAAACCTCTTTCCTTGATGTGCATGGTTCTTTACTTTTGCATCTTACACTCAATTCAAATGAAGAATCTAGATGCTGGCATTTACATATTGAATGTACAAGAGTGTTAAGCAGCAATTCTAGCTCCTTGTGTGTGTGTGTGTGTGTGTGTGTGTGTGTGTGTGCTCAGTTGCTGTCATGTCCAACTCTTTGTGACCCATGGATTTTAGCCCCCCGAGGCTCCTCTGTCCTTGGGATTTTTCCGTAGGATATTGTAAGACACTAGCCTTTGCTGTTTTTCAGAGAACTTGCCATTTCGCCGGGGCTAAATATTGAGTAGAAAGCAGTCTATTGATTAGTGCCTTTAGCTCTAACTATTTAAAGAGTCTCTTGGTTTTGCTTACTTTGTGACAGGGAGAAAACTCTACAATATCATCTTAGAGTGATTTTGTTAGGGGAAGCACACTGACTGAAACTGCCCACCCTGGCCAGGCCCTTTAGTAACCATTTGCATGAGTTATTTTATGACCGGAGATCCTGGTAAGGAATATGGAACTGATAAGCCACCACTAACTGGAAGAGTTCGGGAAAGGTCAAAAGGAGACACCACGTGTCGGTCCACTTCCAAGAATCCCTCTCGCTAGCATCCATCTAGGCTGAGCAATGCGTGCACCACCAGGAAAAACTCTGAATTAGAATGATTGGCCAAAGACCACCCTGAAACTAATCCTATCACCATAAAACCCAAGACTGCAAGCCACATGGCAGAGCAGTTCTCCTGAGTTCCCCTACCCTGCTAATCTCCACCTGGTTGCCCTTTCCCAATAAAATCTCTTGCTTTGTCAGCAGATATGTCTCCTCGGACAATTCATTTCCAAGTGTTAGACAAGAGCCCAGTTTTGGGCTCTGGAAGGGGTCCCCCTTCCTGCAACAATTTCAGAATTTGTGAGTCCTGTTTTCTCAGAGGAGTCATGACACCTAGATTTCAAAACTCAATTCACTTCTCTATTCTTCTCTAATAATTAGTTGTACATGTCACCATTAAAAATAATTTCAATTTCAAATTGTAGCTGCAAGCTATTAAAGGCTTTCTGTCTCTTCCTGCTTTGTTTGTAGTGATCTTGCACTTTATTCTGTTAGGTAGTTGGAATAGGAAAAAGGAGTCCAGCATGGTGGTGGCTAAAAGACAAGGAAGGGAAAAGCCCACTAAAATAGAACAAAGGAAGGTCCAAGGATTGGAATGAGGACCTCAGGTAGAACAAACAGCACTCCTGGCTGGCCCAATTTACACAGGACAAGCCCAGGGGGAGGAGGAAAGACATAGAAAGAGGAGCCAAAGGGCCCAGGCTCTCTGTCTCTTGTCTTTCTCTCTCTTCTTTTTGCATCTTTTGGTTTGGCATGCCCTCACACCTTGAGGATGTATTTTCCTTTATTTTCTAAATAAGACAGCTGTAACACTGGTCTATCTGAGAGCTGTGACGCACTGAGGGCTTTAACGTCTGTCGCTTCAAATTTTTGTTGTGATGAGACAGAACCGAGGAAATTACACACTCCCCTGACAATTCTAATGAAGTAAAATTATCAAAATTATATATCATGAAAAAACCCTCATAAAGTTTAAAGACAGCAGTGCTTGTAACTTACTATTTAAAATTTTATTTATTTTTGTTGTTGTTAATTAATGGAACTGGAATAGACATAAAGACTGTATTCTTTGTGGTGTCATCAATAAAACATTGATTACTTGATTTTTCAAATAAATTTTTGCTTGAACTTTAATGAAGGCATCTCACCATGTTTGAATATTCCCAGCATAGGTTCACAACACATGACTCTGTTTTATTCACATCTCAACTTAGCCAAGTATAAACTGTGTGACTTTGGTTAGTCATTAAACCTCCCTGAACTTCAGCTTCTTTATGTGACATGATACCTAGTCCTTAGGGTTGCTGGATGCACTCTGTTCTGATGGTGTATGTGAAATCTCTAATTTAATGCCTGGAAGAGCCTCAAAAGTGTTCATTTCCTTTCCCTCTGAAGCTGAAGGAAGATGTTTTCTAGAATTTTTTAAATTATGATTTTTAACTTTTAATGGAAAAGAGAAATAAAGACAGAGCAACAGTTTATACAAATTCTCTTCCAGAAATGTGGCAATAGGTATTAGAAACTTCTCTTGTGTCACTTGACCACTAACTTCACATTTTCATGTGGTCACTTGATTAATTTACACAATGAAGTACGTCTCCACAGCTGTCTGTGTATCCCTGGCTGGTAGAAGCCTGTAAACTACACATCCATTTTCAAAGAAGGAAGCCAGCTCCTGTGACCAGGACCTTGACCTCCAAACACCAACTGGGAAATTCCCCATGGTTGGAAGAGCATAGTCCTAACCTTGTGGAAAGTAGTCATCTCTTTAATTTCTCCCCATACCCGAGTACATGTTTGTGAGCCACTGAGGGAGAGACAGTGTGATCCCTGGGAATTATAATACGTCCCACAGCACAGCATCGTTTTGTTGGGGTGTATTATTAGCCTCCAAATTAGAGCCGTGGGGAGCTCCCAACATGGCTTCAGTGGGATGAATAGAGATGAATAAAGCATTCAGCAAAAGGACTTCTGGTGAGATCTAACATAAAGCTGTTTGCTTTGTGCATGTTTAAAATATAGCCTACTACAGCCCTCAAATATATGTAAATTCATCGCTGTCACTCTTTCCTACCCCATGTAAGCCAGTTTACATAATGAATCAGCTCTTACAGCTGTTTTGATGTAGAAATATCACCAACCATTTAAGCTGTGCTGAAGTGACTTATTTTTCCAGGGTTGAAATGGTTTTCTACAGATGGATCACAAGGCTCAGTCATTTTGGCATTGGGAACTGAATCCGTTGTAGCAAAGTGAAATCACAGAGAGAATATCTTTCTATGGTTTCCCCAAGAATAAAAAGAATAATTAAATATTACAGCCCACTGTTCACACATCCATTCATCACCAAATATTGATTAAGGGCCTGTTTCCTCCACTCTCTCCTCCCTTTATCCTTCATGTAGACAGTGACCCTGAAGAGCCTTTGATTTGTCTGTGATGAAAGTGCATCCTATCTGTAAGTTTTTGTGTGAAAATAAATGCCTTTTGAATATATTGAGCACTTTGAGAGAAAGACATCATGCCCTTTCAAATGAACATTATAATAATTTAATAAATTAACTAAACCTGTTCCACTGACACATTGATGAGTCATATGAGTTGCTCTTATCCATTGTTACAAGATTAAACATTTTGGGAATAAAGCTCATCCAATTTATTGAGAAAGAAACTTGGCTACAAATGCAGTCCCACTTAATTAGCTTCTGTTCCAAGTTGCCTAGCTGCTTGAACATAAATGGTGAGTCCCATTGCCCTCTGAGTTTCCTGGGGGCATCTTGCTCTGATCACTTAATTGATTTTCCAAACCAGTTTGTACTCTGGATATATCTTTGTGTCCTTTTTCAGTCTTGTCATTATCATCTTGAAGCATATTCAACAAAATTCTTTGTAGAAGCCCCGTTAGCTATGGAAGAGTGACTGATTAAATTAAGCATATTAAGTACCAACAACAATCCAAACAGGGTGTGGCTGTGTAATGAGTCCTGGTTAGAAGGGAGAGAGGGTGGGATGGTAGTGTGTTTGGTGTTACTGTCAGAGGGACACTCACTGTATCCACGCAAGATGAATGAGGGATGAATTGTGGGCTGGGATGACCAATAGCTTTGAAGCTGTCAACCCACCTCCTTAATCCTGGAACTAAGTACAGTCCAGAAGCCTTTTGACTTAGTTACCAAACTACTAAACTTTCTTTATGAGGATGAAGGCTTTTCAAGAGACAATTAACCTGGAAATTCACAGGAGGTTCTAAGGCAAGCTGGTGTGAGCCAAAGAAGCCAGGAAGGTCAGGGTGGGAGCCCCACCCACCAGCAAGAATGTATAAAAGCTCAGAAGCCTGAAGTGGCATTCACAGTTCAAGAACCAGCCTCAGTGAGTTACCCACATCTCTCCACCAGCACC**ATG**
