# Supplementary material for: Characterization of the Promoter Regions of Two Sheep Keratin-Associated Protein Genes for Hair Cortex-Specific Expression
Source: PLoS One. 2016 Apr 21;11(4):e0153936. doi: 10.1371/journal.pone.0153936 (PMC4839604; doi:10.1371/journal.pone.0153936)
Supplement: S3 Appendix — (DOCX) [file pone.0153936.s003.docx]

1595 CTGAGGGAGA GACAGTGTGA TCCCTGGGAA TTATAATACG TCCCACAGCA CAGCATCGTT

**A**

**NF-KappaB(p65)**

1535 TTGTTGGGGT GTATTATTAG CCTCCAAATT AGAGCCGTG**G GGA**GCTCCCA ACATGGCTTC

**GATA3**

1475 AGTGGGATGA ATAGAGATGA ATAAAGCATT CAGCAAAAGG ACTTCTGGTG A**GATCT**AACA

1415 TAAAGCTGTT TGCTTTGTGC ATGTTTAAAA TATAGCCTAC TACAGCCCTC AAATATATGT

1355 AAATTCATCG CTGTCACTCT TTCCTACCCC ATGTAAGCCA GTTTACATAA TGAATCAGCT

1295 CTTACAGCTG TTTTGATGTA GAAATATCAC CAACCATTTA AGCTGTGCTG AAGTGACTTA

**AP-1**

1235 TTTTTCCAGG GTTGAAATGG TTTTCTACAG ATGGATCACA AGGCTC**AGTC A**TTTTGGCAT

1175 TGGGAACTGA ATCCGTTGTA GCAAAGTGAA ATCACAGAGA GAATATCTTT CTATGGTTTC

**Cart-1**

1115 CCCAAGAATA AAAAGAAT**AA TTA**AATATTA CAGCCCACTG TTCACACATC CATTCATCAC

1055 CAAATATTGA TTAAGGGCCT GTTTCCTCCA CTCTCTCCTC CCTTTATCCT TCATGTAGAC

995 AGTGACCCTG AAGAGCCTTT GATTTGTCTG TGATGAAAGT GCATCCTATC TGTAAGTTTT

935 TGTGTGAAAA TAAATGCCTT TTGAATATAT TGAGCACTTT GAGAGAAAGA CATCATGCCC

**Oct-1**

875 TTTCAAATGA ACATTATAA**T AATT**TAATAA ATTAACTAAA CCTGTTCCAC TGACACATTG

**AP-1**

815 ATG**AGTC**ATA TGAGTTGCTC TTATCCATTG TTACAAGATT AAACATTTTG GGAATAAAGC

755 TCATCCAATT TATTGAGAAA GAAACTTGGC TACAAATGCA GTCCCACTTA ATTAGCTTCT

**NF-KappaB(p65)**

695 GTTCCAAGTT GCCTAGCTGC TTGAACATAA ATGGTGAGTC CCATTGCCCT CTGAG**TTTCC**

**Cart-1**

635 TGGGGGCATC TTGCTCTGAT CACT**TAATT**G ATTTTCCAAA CCAGTTTGTA CTCTGGATAT

575 ATCTTTGTGT CCTTTTTCAG TCTTGTCATT ATCATCTTGA AGCATATTCA ACAAAATTCT

515 TTGTAGAAGC CCCGTTAGCT ATGGAAGAGT GACTGATTAA ATTAAGCATA TTAAGTACCA

455 ACAACAATCC AAACAGGGTG TGGCTGTGTA ATGAGTCCTG GTTAGAAGGG AGAGAGGGTG

395 GGATGGTAGT GTGTTTGGTG TTACTGTCAG AGGGACACTC ACTGTATCCA CGCAAGATGA

**CCAAT**

335 ATGAGGGATG AATTGTGGGC TGGGATGA**CC AAT**AGCTTTG AAGCTGTCAA CCCACCTCCT

275 TAATCCTGGA ACTAAGTACA GTCCAGAAGC CTTTTGACTT AGTTACCAAA CTACTAAACT

215 TTCTTTATGA GGATGAAGGC TTTTCAAGAG ACAATTAACC TGGAAATTCA CAGGAGGTTC

155 TAAGGCAAGC TGGTGTGAGC CAAAGAAGCC AGGAAGGTCA GGGTGGGAGC CCCACCCACC

**TATA**

95 AGCAAGAATG **TATAAAA**GCT CAGAAGCCTG AAGTGGCATT CACAGTTCAA GAACCAGCCT

**+1**

**5’UTR**

35 CAGTGAGTTA CCCACATCTC TCCACCAGCA CC**ATG**

1595 CTGAGGGAGA GACAGTGTGA TCCCTGGGAA TTATAATACG TCCCACAGCA CAGCATCGTT

**B**

**NF-KappaB(p65)**

1535 TTGTTGGGGT GTATTATTAG CCTCCAAATT AGAGCCGTG**G GGA**GCTCCCA ACATGGCTTC

**GATA3**

1475 AGTGGGATGA ATAGAGATGA ATAAAGCATT CAGCAAAAGG ACTTCTGGTG A**GAT**CTAACA

1415 TAAAGCTGTT TGCTTTGTGC ATGTTTAAAA TATAGCCTAC TACAGCCCTC AAATATATGT

1355 AAATTCATCG CTGTCACTCT TTCCTACCCC ATGTAAGCCA GTTTACATAA TGAATCAGCT

1295 CTTACAGCTG TTTTGATGTA GAAATATCAC CAACCATTTA AGCTGTGCTG AAGTGACTTA

**AP-1**

1235 TTTTTCCAGG GTTGAAATGG TTTTCTACAG ATGGATCACA AGGCTCA**GTC A**TTTTGGCAT

1175 TGGGAACTGA ATCCGTTGTA GCAAAGTGAA ATCACAGAGA GAATATCTTT CTATGGTTTC

**Cart-1**

1115 CCCAAGAATA AAAAGAA**TAA TT**AAATATTA CAGCCCACTG TTCACACATC CATTCATCAC

1055 CAAATATTGA TTAAGGGCCT GTTTCCTCCA CTCTCTCCTC CCTTTATCCT TCATGTAGAC

995 AGTGACCCTG AAGAGCCTTT GATTTGTCTG TGATGAAAGT GCATCCTATC TGTAAGTTTT

935 TGTGTGAAAA TAAATGCCTT TTGAATATAT TGAGCACTTT GAGAGAAAGA CATCATGCCC

**OCT-1**

875 TTTCAAATGA ACATTATAAT **AATT**TAATAA ATTAACTAAA CCTGTTCCAC TGACACATTG

**AP-1**

815 ATGA**GTCA**TA TGAGTTGCTC TTATCCATTG TTACAAGATT AAACATTTTG GGAATAAAGC

755 TCATCCAATT TATTGAGAAA GAAACTTGGC TACAAATGCA GTCCCACTTA ATTAGCTTCT

**NF-KappaB(p65)**

695 GTTCCAAGTT GCCTAGCTGC TTGAACATAA ATGGTGAGTC CCATTGCCCT CTGAGT**TTCC**

**Cart-1**

635 TGGGGGCATC TTGCTCTGAT CACT**TAAT**TG ATTTTCCAAA CCAGTTTGTA CTCTGGATAT

575 ATCTTTGTGT CCTTTTTCAG TCTTGTCATT ATCATCTTGA AGCATATTCA ACAAAATTCT

515 TTGTAGAAGC CCCGTTAGCT ATGGAAGAGT GACTGATTAA ATTAAGCATA TTAAGTACCA

455 ACAACAATCC AAACAGGGTG TGGCTGTGTA ATGAGTCCTG GTTAGAAGGG AGAGAGGGTG

395 GGATGGTAGT GTGTTTGGTG TTACTGTCAG AGGGACACTC ACTGTATCCA CGCAAGATGA

**CCAAT**

335 ATGAGGGATG AATTGTGGGC TGGGATGA**CC AAT**AGCTTTG AAGCTGTCAA CCCACCTCCT

275 TAATCCTGGA ACTAAGTACA GTCCAGAAGC CTTTTGACTT AGTTACCAAA CTACTAAACT

215 TTCTTTATGA GGATGAAGGC TTTTCAAGAG ACAATTAACC TGGAAATTCA CAGGAGGTTC

155 TAAGGCAAGC TGGTGTGAGC CAAAGAAGCC AGGAAGGTCA GGGTGGGAGC CCCACCCACC

**TATA**

95 AGCAAGAATG **TATAAAA**GCT CAGAAGCCTG AAGTGGCATT CACAGTTCAA GAACCAGCCT

**+1**

**5’UTR**

35 CAGTGAGTTA CCCACATCTC TCCACCAGCA CC**ATG**

**S3 Appendix. Transcription factor binding sites predicted by TRANSFAC (A) and MatInspector**  **(B).** The consensus binding sites of NF-KappaB(p65), GATA3, AP-1, Cat-1 and Oct-1 were predicated by the two different programs and are shown by *shadow boxes*. The core promoter which is shown by *black box* was predicated by promoter scan and promoter 2.0 programs. The underlined sequence is the 5’ UTR of the sheep *KRTAP11-1* gene, and the A (the arrow indicated) is the transcriptional start site.
